# Loss of ficolin-3 expression is associated with poor prognosis in patients with hepatocellular carcinoma

**DOI:** 10.7150/ijms.84729

**Published:** 2023-07-03

**Authors:** Chia-Chi Chen, Teng-Hung Yu, Cheng-Ching Wu, Wei-Chin Hung, Thung-Lip Lee, Wei-Hua Tang, I-Ting Tsai, Fu-Mei Chung, Yau-Jiunn Lee, Chia-Chang Hsu

**Affiliations:** 1 Department of Pathology, E-Da Hospital, I-Shou University, Kaohsiung 82445 Taiwan.; 2 The School of Chinese Medicine for Post Baccalaureate, College of Medicine, I-Shou University, Kaohsiung 82445 Taiwan.; 3 Department of Physical Therapy, I-Shou University, Kaohsiung 82445 Taiwan.; 4 Department of Occupational therapy, I-Shou University, Kaohsiung 82445 Taiwan.; 5 Division of Cardiology, Department of Internal Medicine, E-Da Hospital, I-Shou University, Kaohsiung 82445 Taiwan.; 6 School of Medicine, College of Medicine, I-Shou University, Kaohsiung 82445 Taiwan.; 7 Division of Cardiology, Department of Internal Medicine, E-Da Cancer Hospital, I-Shou University, Kaohsiung 82445 Taiwan.; 8 School of Medicine for International Students, College of Medicine, I-Shou University, Kaohsiung 82445 Taiwan.; 9 Division of Cardiology, Department of Internal Medicine, Taipei Veterans General Hospital, Yuli Branch, Hualien 98142 Taiwan.; 10 Faculty of Medicine, School of Medicine, National Yang Ming Chiao Tung University, Taipei 112304 Taiwan.; 11 Department of Emergency, E-Da Hospital, I-Shou University, Kaohsiung, 82445 Taiwan.; 12 Lee's Endocrinologic Clinic, Pingtung 90000 Taiwan.; 13 Division of Gastroenterology and Hepatology, Department of Internal Medicine, E-Da Hospital, I-Shou University, Kaohsiung 82445 Taiwan.; 14 Health Examination Center, E-Da Dachang Hospital, I-Shou University, Kaohsiung 80794 Taiwan.

**Keywords:** Hepatocellular carcinoma, ficolin-3, overall survival, immunohistochemistry

## Abstract

**Background:** Ficolin-3 (FCN3) is a well-known circulating pattern recognition molecule which plays a role in host immune responses to cancer via activation of the lectin complement pathway. Nevertheless, the clinical significance of FCN3 in patients with hepatocellular carcinoma (HCC) is unclear.

**Methods:** Eighty-seven HCC patients who received hepatectomy at our hospital were included. Immunohistochemical staining was used to assess the FCN3 expression in both tumorous and non-tumorous tissues from the patients, who were classified into high and low expression groups. Differences in clinicopathological characteristics between the two groups were then analyzed.

**Results:** Survival was significantly associated with FCN3 immunohistochemical score (p for trend = 0.048). Kaplan-Meier analysis revealed a higher overall survival rate in the patients with a high FCN3 expression than in those with a low FCN3 expression (p=0.031). A high FCN3 expression in tumor tissue was independently associated with better overall survival (p=0.042). However, multivariate analysis showed that FCN3 expression was not an independent risk factor for overall survival.

**Conclusion:** Our findings suggest that FCN3 is significantly related to the prognosis of HCC. FCN3 may be a prognostic marker in patients with HCC.

## Introduction

Liver cancer is the sixth most prevalent cancer and the third most fatal cancer (after lung and colorectal cancer) worldwide, accounting for about 906,000 newly diagnosed cases and 8.3% of all cancer-related deaths 1. Among all primary liver tumors, hepatocellular carcinoma (HCC) is the most predominant histopathological subtype (approximately 75%-85%), and the associated morbidity, disability, and mortality are important health issues and economic burden for the patients, their family and society. Many risk factors are associated with the tumorigenesis of HCC, including chronic viral hepatitis, liver cirrhosis, toxins, smoking, alcohol consumption, obesity, and diabetes 2. Although there are many treatment options for HCC in international guidelines, including surgery, arterially directed therapies, ablation, chemotherapy, radiotherapy, targeted therapy, immunotherapy, and transplantation 3,4, due to molecular and phenotypic heterogeneity, the recurrence rate is still high and the overall survival rate is poor, especially in those with metastatic disease 5. Since both epigenetic and genetic alterations are involved in the carcinogenesis and progression of HCC, understanding these alterations can help to elucidate the molecular mechanisms underlying hepato-carcinogenesis and also the prognosis 6.

The ficolin (FCN) family of proteins in humans is comprised of three members, FCN1, FCN2, and FCN3, and they participate in activating the lectin complement pathway by binding their mannose-binding lectin (MBL)-like structure to proteins of the MBL-associated serine proteases (MASP) family, and therefore act as tumor suppressors. FCNs have been identified in many different tissues and been shown to contribute to tissue hemostasis, with FCN1 primarily existing inside immune cells and FCN2 and FCN3 mainly presenting in the serum 7,8. FCN3 (H-ficolin, Hakata antigen), coded by the FCN3 gene localized on 1p36.11, is composed of several subunits each containing three C-terminal recognition domains and a collagen-like strand binding to acetyl groups on microbial surfaces. High expressions of FCN3 have been demonstrated in the liver and lungs, with the liver assumed to account for circulating levels 9. Previously, a lower expression of FCN3 has been found and proved either in tissue or serum from numerous cancer patients such as ovarian cancer, multiple myeloma, lymphoma, acute myeloid leukemia, head and neck cancer, and lung cancer 8,10-15. However, few studies have investigated differences in the expression of FCN3 between patients with HCC with/without hepatitis viral infection and normal tissue, and the possible role of FCN3 in O-GlcNAcylation recognition in bioinformatic, cell line, RNA, and serum experiments 16-18. In addition, relationships between FCN3 expression and clinicopathological characteristics in patients with HCC have yet to be elucidated.

Therefore, this study aimed to investigate the expression pattern of FCN3 in patients with HCC using immunohistochemistry (IHC) in tumorous and non-tumorous tissues. We also investigated the relationship between the clinicopathological characteristics and FCN3 expression in patients with HCC, and whether FCN3 could be a prognostic biomarker in these patients.

## Methods

### Study subjects

We enrolled 87 patients with HCC who received resection for HCC from January 2015 to December 2017. Those receiving any other treatment were excluded. The Institutional Review Board of E-Da Hospital approved the study (no. EMRP-109-108) and all patients provided written informed consent. The medical records of the hospital were used to extract information on the patients. Of the enrolled patients, 19 were women and 68 were men, with a mean age of 62 (range, 38 to 88) years. Patients with advanced stage or recurrent of HCC, nor metastatic liver tumor were not included in this study. Staging was based on the American Joint Committee on Cancer. All patients received regular follow-up at our outpatient clinic and received prospective standard examinations for recurrence. Resected tumorous and non-tumorous tissue specimens of the liver were kept at -80˚C for subsequent assay.

### FCN3 Expression Analysis

For immunohistochemical studies, the tissue samples were obtained from patients with newly diagnosed operable HCC who received tumor resection. An automated Bond-Max system (Leica Microsystems) was used for FCN3 immunostaining. In brief, tissue sections cut from formalin-fixed, paraffin-embedded tissue microarray blocks were placed on slides and allowed to dry for 1 hour at 60°C. Bond Universal Covertiles were then placed on the slides and analyzed using the Bond-Max system (Leica Microsystems) according to the manufacturer's protocol as follows: (a) The tissue sections were deparaffinized by rinsing with Bond Dewax Solution at 72°C; (b) Heat-induced epitope retrieval (antigen unmasking) was performed using Bond Epitope Retrieval Solution 2 for 40 minutes at 100°C; (c) Peroxide block was added to the slides for 5 minutes at room temperature; (d) The slides were incubated with mouse monoclonal anti-FCN3 (Proteintech. 11867-1-AP) antibodies at a dilution of 1:100 for 120 minutes at room temperature; (e) Bond Polymer was added to the slides for 8 minutes at room temperature; (f) Color was developed using 3, 3'- diaminobenzidine tetrahydrochloride (DAB) as the chromogen for 5 minutes at room temperature; and (g) Counterstaining was performed using hematoxylin for 5 minutes, after which the slides were mounted and examined using light microscopy. In addition, among 87 patients with HCC, we performed immunohistochemical analysis of 33 pairs of HCC and non-tumor tissues. The results of FCN3 expression levels between tumor and non-tumor tissues were defined according to immunohistochemical staining. The intensity of immunostaining was graded as “0” for no immunostaining, 1+ for weak but definitely detectable immunostaining, 2+ for moderate immunostaining, and 3+ or 4+ for strong immunostaining.

### Immunohistochemical Staining

The results of FCN3 IHC staining were scored from 1 to 4 based on the percentages of cells with positive staining as follows: <25%, 25-50%, 51-75%, and >75% positive cells, respectively. Two independent researchers evaluated all scores separately at the same time and under the same conditions. Cases of disagreement were resolved by consensus.

### Statistical Analyses

JMP version 10.0 for Windows (SAS Institute, Cary, NC, USA) was used for all statistical analyses. Categorical variables were presented as frequency (percentage), and differences were analyzed using the Fisher's exact test. The prevalence of survival status and trends in the four FCN3 IHC score groups were analyzed using the Fisher's exact test and Cochran-Armitage trend test respectably. Survival was assessed using the Kaplan-Meier method, with differences between groups analyzed using the log-rank test. Hazard ratios (HRs) and corresponding 95% confidence intervals (CI) were calculated using univariate and multivariate Cox proportional hazard models to determine the factors independently associated with survival. A p-value less than 0.05 was considered statistically significant.

## Results

### Correlation between FCN3 expression and clinicopathological characteristics

**Figure 1:**
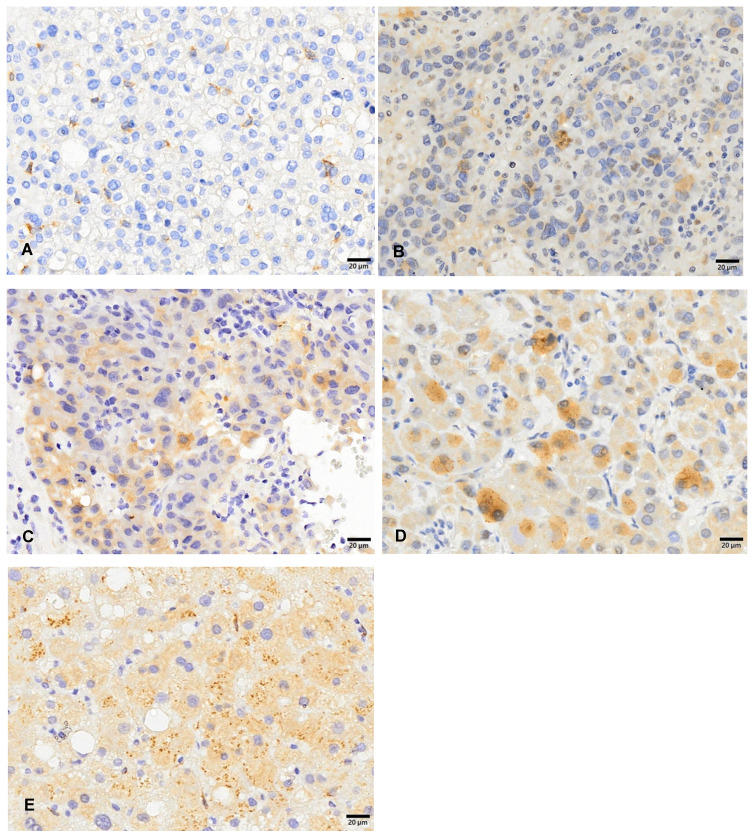
Cytoplasmic ficolin-3 immunoreactivity in hepatocellular carcinoma tissue specimens. Immunoreactivity was analyzed using a 4-tier grading system: grade 1 (A), grade 2 (B), grade 3 (C), grade 4 (D), and non-tumor tissue (E).

FCN3 immunoreactivity was evaluated using the following system: grade 1 (A), grade 2 (B), grade 3 (C), and grade 4 (D). The expression of FCN3 was detected in the cytoplasm or both cytoplasm and partial nuclear areas of all HCC tissue specimens (Figure 1). The FCN3 IHC results showed a higher expression of FCN3 in the normal tissue specimen (Figure 1E). In addition, among 33 pairs of HCC and non-tumor tissues, FCN3 expression levels showed a consistency strong immunostaining (3+ to 4+) in non-tumor tissues (Table 1). This suggested that there may be lower levels of FCN3 in HCC tissues, and that FCN3 may be a tumor suppressor.

**Figure 2:**
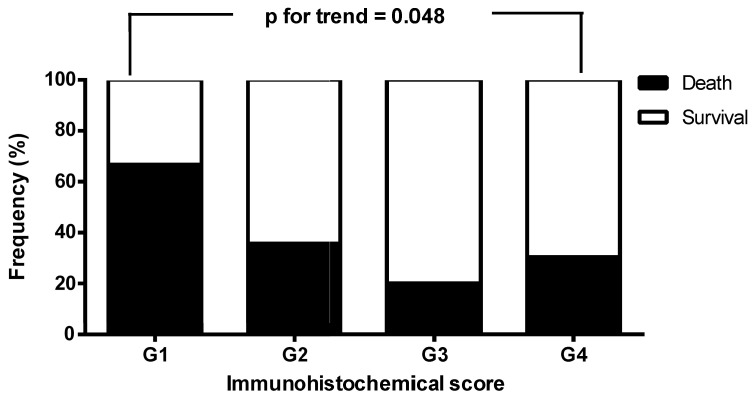
The frequencies of death and survival of the patients with hepatocellular carcinoma stratified by ficolin-3 (FCN3) immunohistochemical (IHC) score. Survival status was significantly associated with FCN3 IHC score.

We next investigated the clinicopathological variables of the patients according to FCN3 expression levels in the tumor tissue specimens. The patients with FCN3 grade 3 and 4 were associated with better survival than those with FCN3 grade 1 and 2 (p=0.047, Table 2). However, there were no significant differences in sex, age, alcohol drinking, tumor size, etiology, pathological differentiation, TNM stage, and early recurrence between the two groups (Table 2). We then analyzed the frequencies of death and survival of the patients with HCC stratified by FCN3 IHC score, and found that survival status was significantly associated with FCN3 IHC score (survival vs. death, G1/G2/G3/G4 = 33.3%/ 64.3%/80.0%/ 69.6% vs. 66.7%/35.7%/ 20.0%/30.4%; p for trend = 0.048, Figure 2).

### Association between FCN3 expressions and overall survival

**Figure 3:**
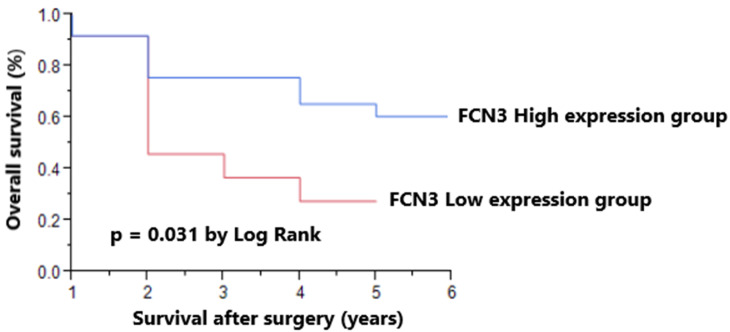
Overall survival in patients with low and high expressions of ficolin-3 (FCN3) in tumor tissue. The overall survival rate was significantly higher in the group with a high FCN3 expression.

We next examined overall survival in the patients with high and low FCN3 expressions, and found that the group with a high FCN3 expression had a significantly higher overall survival rate (Figure 3). Univariate analysis showed that a low FCN3 expression, age ≥65 years, and poor differentiation were significant prognostic indicators of poor overall survival (Table 3). Multivariate analysis showed that age ≥65 years and poor differentiation but not sex, hepatitis B/C virus (HBV/HCV) infections, tumor size, stage, and FCN3 expression were independent prognostic indicators in the HCC patients (Table 4).

## Discussion

In this study, we analyzed the association between FCN3 and HCC and identified three main findings. First, the patients with FCN3 IHC grade 3 and 4 were associated with better survival than those with FCN3 IHC grade 1 or 2. Second, univariate analysis showed that a low expression of FCN3, age ≥65 years, and poor differentiation were significantly associated with poor overall survival. Third, the patients with a high FCN3 expression had significantly better overall survival.

Our findings of significantly better overall survival in the patients with a high expression of FCN3 in tumor tissues are consistent with previous studies 7,17,19-21. Lai et al. reported a lower expression of FCN3 in HCC specimens 19, and Wang et al. reported that a lower FCN3 expression was associated with worse overall survival in patients with HCC 20. In addition, Lin et al. suggested that a lower expression of FCN3 in tumor tissues may be associated with HCC tumorigenesis and venous metastasis, and that a high expression may be a favorable prognostic indicator in patients with HCC 21.

FCNs play roles in environmental homeostasis and innate immunity in tissue. They are composed of at least four linked trimers, and they are structurally similar to MBL. The domains with a collagen-like structure interact with MASPs to create complexes associated with activation of the lectin complement pathway 22, which is one of the three methods of complement activation 23. Correlations between FCN genes with liver, lung, and ovarian cancers have been reported, indicating that there may be a link between FCN genes and tumorigenesis 10,24. Therefore, proteins that activate complement may both contribute to the development of cancer and also affect the course of the underlying disease.

FCN3 has been shown to be epigenetically silenced in various tumors 7,10,17, 19,20, suggesting that abnormally expressed FCN3 may participate in activation of the lectin complement pathway during tumor development. Sun et al. reported an association between FCN3 and immune cell infiltration in patients with HCC, and immune cells have been associated with the development and progression of HCC 25. However, further studies are needed to investigate the association between the expression patterns of FCN3 in HCC tissues and tumor progression.

The overexpression of FCN3 has been shown to inhibit cell proliferation and induce apoptosis via the p53 signaling pathway 26. A proposed mechanism is that FCN3 modulates the nuclear translocation of eukaryotic initiation factor 6 (EIF6) by binding to ribosome maturation factor (SBDS), which then leads to activation of the p53 pathway. In addition, Y-box binding protein 1 (YBX1) has been shown to regulate the translation and transcription of FCN3 to SBDS 26. These results indicate that the FCN3/YBX1/SBDS axis may be a novel therapeutic target for HCC. We did not assess the underlying molecular/cellular mechanisms of FCN3 in HCC tumorigenesis in this study, and further studies are warranted to investigate this issue.

HBV/HCV infections can induce genetic instability and have been shown to be important risk factors for HCC 27,28. Several studies have suggested associations between serum FCN3 and FCN2 levels with HCV and HBV infections in patients with HCC 29,30. However, we found no significant differences in the expression of FCN3 in the HCC patients with HBV and HCV infection (Table 2). This may be due to the use of patient serum versus patient tissues.

As mentioned, previous studies have shown longer overall survival in patients with various cancers and a high FCN3 expression level 7,10,19,31. In this study, we assessed the prognostic role of FCN3 for HCC using survival analysis based on the FCN3 expression in tumor tissues, and found that the patients with a low FCN3 expression had significantly worse overall survival in univariate analysis. However, FCN3 expression was not an independent prognostic factor for HCC in multivariate analysis, and there was no significant association with disease-free survival.

A potential limitation of this study is that we only used IHC, and further studies are needed to elucidate the role of FCN3 in HCC. Furthermore, we used anonymized pathological data from our hospital, and thus we could not obtain matched tissue and blood samples to perform further analysis after anonymization. As a result, we did not present the results of mechanistic molecular studies such as qPCR and Western blot in this report. Moreover, in the present study, patients with advanced stage or recurrent of HCC, nor metastatic liver tumor were not included in this study. Hence, we cannot calculate the expression levels of FCN3 between early vs. recurrent/metastatic stages. Further investigations are needed to investigate whether tumor tissues may have different expression levels in early vs. recurrent/metastatic stages.

## Conclusions

The results of this study suggest that FCN3 may play a role in repressing the development of HCC, at least to some degree, and that FCN3 may be a promising prognostic indicator in patients with HCC.

## Figures and Tables

**Table 1:** Immunohistochemical staining for FCN3 in tumor and non-tumor tissues

Intensity of immunostaining	Tumor	Non-tumor
No	33	33
0	1(3.0)	0(0.0)
1+	7(21.2)	0(0.0)
2+	13(39.4)	0(0.0)
3+	12(36.4)	0(0.0)
3+~4+	0(0.0)	20(60.6)
4+	0(0.0)	13(39.4)

The intensity of immunostaining was graded as “0” for no immunostaining, 1+ for weak but definitely detectable immunostaining, 2+ for moderate immunostaining, and 3+ or 4+ for strong immunostaining.

**Table 2:** Correlation between ficolin-3 expression and clinicopathological characteristics

Clinical parameter	All cases	FCN3 grade 1, 2 N (%)	FCN3 grade 3, 4 N (%)	p-value
No	87	26	61	
Sex				
Male	68(78.2)	21(80.8)	47(77.1)	0.784
Female	19(21.8)	5(19.2)	14(23.0)	
Age (yr)				
<65	11(12.6)	15(57.7)	40(65.6)	0.485
≥65	76(87.4)	11(42.3)	21(34.4)	
Alcohol drinking				
Yes	53(60.9)	13(50.0)	40(65.6)	0.173
No	34(39.1)	13(50.0)	21(34.4)	
Tumor size (cm)				
<5	58(66.7)	14(53.9)	44(72.1)	0.098
≥5	29(33.3)	12(46.2)	17(27.9)	
Etiology				
HCC	17(19.5)	5(19.2)	12(19.7)	0.962
HCC/HBV	43(49.4)	14(53.9)	29(47.5)	0.590
HCC/HCV	24(27.6)	7(26.9)	17(27.9)	0.928
HCC/HBV/HCV	3(3.5)	0(0.0)	3(4.9)	0.250
Pathological differentiation				
Well	15(17.2)	2(7.7)	13(21.3)	0.124
Moderately	65(74.7)	20(76.9)	45(73.8)	0.757
Poorly	7(8.1)	4(15.4)	3(4.9)	0.100
TNM stage				
I	50(57.5)	12(46.2)	38(62.3)	0.236
II	21(24.1)	8(30.8)	13(21.3)	0.414
IIIa-IVb	16(18.4)	6(23.1)	10(16.4)	0.548
Early recurrence				
Absent	35(40.2)	10(38.5)	25(41.0)	0.826
Occurs	52(59.8)	16(61.5)	36(59.0)	
Survival status				
Survival	57(65.5)	13(50.0)	44(72.1)	**0.047**
Death	30(34.5)	13(50.0)	17(27.9)	

FCN3, ficolin-3; HCC, hepatocellular carcinoma; HBV, hepatitis B virus; HCV, hepatitis C virus.

**Table 3:** Univariate analysis of factors affecting overall survival in the patients with hepatocellular carcinoma

Variable	Hazard ratio	95% CI	p-value
Age: ≥65 years versus <65 years	2.801	1.347-6.120	**0.006**
Sex: Male versus female	1.477	0.611-4.391	0.409
Differentiation status:			
Poorly versus well and moderately	3.256	1.183-7.709	**0.025**
Hepatitis B Virus: Positive versus negative	0.569	0.261-1.179	0.130
Hepatitis C Virus: Positive versus negative	1.793	0.851-3.691	0.122
Tumor size: >5 cm versus ≤5 cm	1.444	0.687-2.961	0.325
Stage: 3, 4 versus 1, 2	1.608	0.669-3.496	0.272
Ficolin-3 in tumor: High versus low	0.345	0.203-1.076	**0.042**

**Table 4:** Multivariate analysis of factors affecting overall survival in the patients with hepatocellular carcinoma using a Cox proportional hazard model

Variable	Hazard ratio	95% CI	p-value
Age: ≥65 years versus <65 years	2.702	1.128-6.819	**0.025**
Sex: Male versus female	0.895	0.304-3.038	0.848
Differentiation status:			
Poorly versus well and moderately	3.923	1.262-11.191	**0.020**
Hepatitis B Virus: Positive versus negative	0.590	0.210-1.800	0.338
Hepatitis C Virus: Positive versus negative	0.992	0.371-2.926	0.987
Tumor size: >5 cm versus ≤5 cm	1.460	0.472-4.170	0.497
Stage: 3, 4 versus 1, 2	1.810	0.604-5.546	0.287
Ficolin-3 in tumor: High versus low	0.623	0.262-1.613	0.315

In multivariate Cox regression analysis, values included for analysis were age, sex, differentiation status, hepatitis B Virus, hepatitis C Virus, tumor size, stage, and ficolin-3 in tumor.
